# Microbial conversion of biodiesel waste for carotenoid production

**DOI:** 10.3389/fbioe.2026.1851919

**Published:** 2026-06-29

**Authors:** Katherine R. Weber, Zoe Chu, Haley Desai, Nicholas Cox, Dragan Simovic, Julie A. Maupin-Furlow

**Affiliations:** 1 Department of Microbiology and Cell Science, Institute of Food and Agricultural Science, University of Florida, Gainesville, FL, United States; 2 Lola Vega, Hollywood, FL, United States; 3 Genetics Institute, University of Florida, Gainesville, FL, United States

**Keywords:** archaea, biotechnology, carotenoid biosynthesis, crude glycerin, *Haloferax volcanii*, urea

## Abstract

**Introduction:**

Enhanced production of isoprenoid compounds, including carotenoids, is needed to meet the growing demand in the food, cosmetic, pharmaceutical, and biotechnology sectors. *Haloferax volcanii* represents a promising microbial platform for sustainable isoprenoid production, as this halophilic archaeon is well suited for metabolic engineering, thrives under harsh conditions (*e.g.,* UV irradiation, high temperatures, and metal-induced stress) compatible with bioprocessing, and naturally synthesizes carotenoids including the high-value C_50_ bacterioruberin.

**Methods:**

In this study, we optimized carotenoid yield in *H. volcanii* using a chemically defined medium supplemented with cost-effective, industrially favorable feedstocks of crude glycerin and urea as the sole carbon and nitrogen sources, respectively. Following optimization by reuse of medium and supplementation with additional glycerin, carotenoid production was evaluated. Transcript abundance of the carotenoid 3,4-desaturase gene (*crtD*, HVO_2528) was measured, and β-galactosidase (*bga*H) reporter activity was used to assess *crtD* promoter activity.

**Results:**

*H. volcanii* was found to display comparable growth rates on crude glycerin to laboratory grade glycerol. Following optimization by reuse of medium and supplementation with additional glycerin, an 8-fold increase in carotenoid yield was observed when urea (84.1 ± 8.4 mg⋅L^−1^) served as the nitrogen source compared to cultures only grown with crude glycerin and NH_4_Cl (10.0 ± 2.4 mg⋅L^−1^) when normalized. The higher carotenoid yield on urea vs. NH_4_Cl was found to be correlated with a 3- to 4-fold increase in transcript abundance of the carotenoid 3,4-desaturase gene (*crtD*, HVO_2528) that was regulated at the level of transcription. The *crtD* promoter was therefore identified as a strong candidate based on β-galactosidase (*bga*H) reporter activity for use in metabolic engineering.

**Discussion:**

Urea offers higher nitrogen content, reduced acidification potential, and greater scalability than NH_4_Cl for bioprocessing applications. Together, our findings support the development of a sustainable, circular approach for repurposing industrial glycerin waste streams to support carotenoid production using urea as a nitrogen source and *H. volcanii* as a microbial biocatalyst for renewable biomanufacturing.

## Introduction

Carotenoids are a diverse class of naturally occurring bioactive pigments that play essential roles in photoprotection, antioxidant defense, and cellular stability across a wide range of organisms (Guedes et al., 2011; Sandmann, 2025; Vílchez et al., 2011). To date, 1,117 carotenoids have been identified (Yabuzaki, 2017), reflecting their extensive structural diversity and broad functional significance in both biological and industrial contexts. Carotenoids have attracted considerable interest due to their chemical versatility and diverse applications across various industries. In nutraceutical and pharmaceutical contexts, carotenoids function as potent antioxidants that neutralize free radicals and mitigate oxidative stress (Zalazar et al., 2019). Their incorporation into animal feed improves livestock health and productivity by supplying essential nutrients and antioxidant protection (Yunitasari et al., 2023). Carotenoids are also widely used as natural colorants in food and cosmetic products, offering safe alternatives to synthetic additives while providing protection against UV radiation and environmental stressors (Baswan et al., 2020; Calegari-Santos et al., 2016; Cojoc et al., 2019). Their bioactive properties, including anti-inflammatory and anticancer activities, are actively explored for therapeutic development, and their distinctive pigmentation enables use as visual markers in biotechnological applications (Couillard et al., 2016; Teodoro et al., 2012). Together, these functional and bioactive characteristics position carotenoids as high-value compounds with broad industrial relevance.

Among the identified carotenoids, bacterioruberin is a major carotenoid common in halophilic, or salt loving, organisms. The accumulation of this C_50_ carotenoid is responsible for the characteristic pink–red pigmentation of the microorganism and contributes to cellular photoprotection properties (Jehlička et al., 2013; Peck et al., 2019). Bacterioruberin differs from more common carotenoids by possessing four hydroxyl groups and an extended system of conjugated double bonds, features that contribute to its enhanced bioactivity (Delgado-Garcia et al., 2023). In addition to bacterioruberin, halophilic archaea synthesize a range of carotenoids, including lower concentrations of bacterioruberin derivatives, phytoene, lycopene, and salinixanthin. However, bacterioruberin represents the predominant carotenoid produced by haloarchaeal species (de Lourdes Moreno et al., 2012; Jehlička et al., 2013; Lin et al., 2025; Palanisamy and Ramalingam, 2024; Yatsunami et al., 2014). Carotenoids are widely associated with protection against oxidative stress and contribute to survival under extreme or fluctuating environmental conditions (Giani and Martínez-Espinosa, 2020; Lim et al., 2023; Othman et al., 2017; Saini et al., 2019; Sandmann, 2019). Consistent with this, bacterioruberin production has also been reported in diverse non-model microorganisms isolated from environmental source, including *Arthrobacter agilis* (Noby et al., 2023) *Micrococcus roseus* (Strand et al., 1997), *Rubrobacter radiotolerans* (Saito et al., 1994), and *Salinicoccus roseus* (Kesbiç and Gültepe, 2023).

**FIGURE 1:**
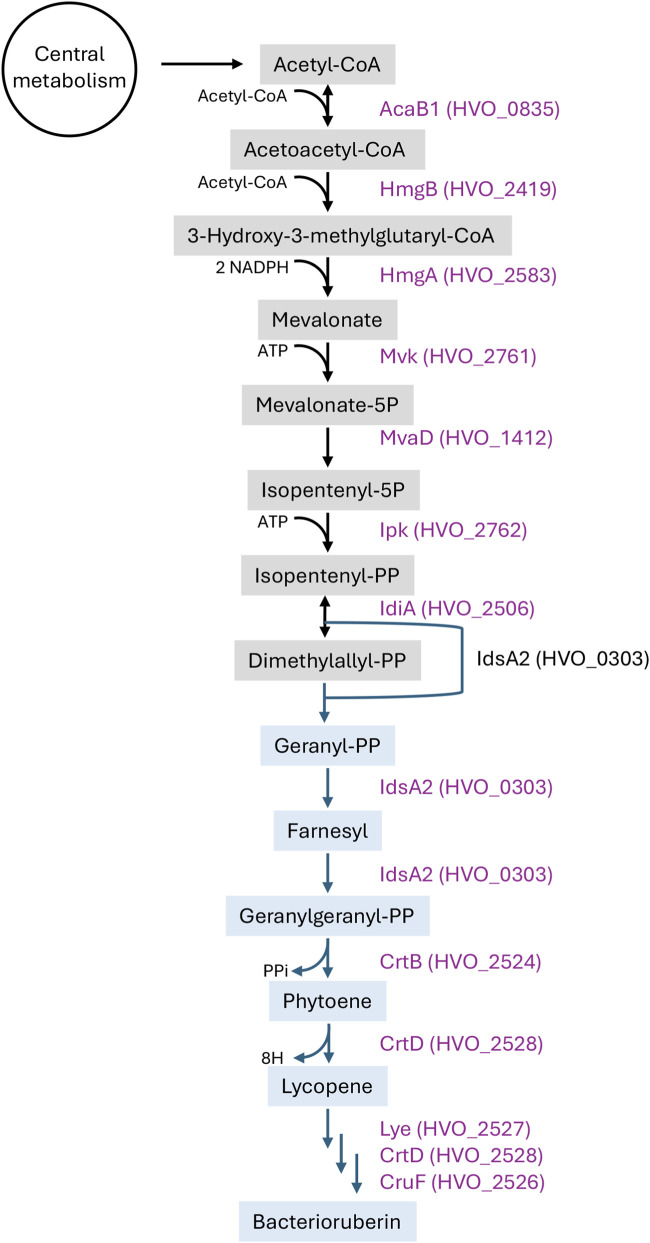
Proposed pathways of mevalonate and bacterioruberin biosynthesis in *H. volcanii*. Steps of mevalonate (grey rectangles) and bacterioruberin (blue rectangle) biosynthesis adapted from KEGG (Kanehisa et al., 2025). Enzyme and gene locus tag number indicated on right. In brief, acetyl-CoA from central metabolism enters the mevalonate pathway to supply the isoprenoid precursors. Two molecules of acetyl-CoA form acetoacetyl-CoA by acetyl-CoA C-acetyltransferase (AcaB1, HVO_0835), followed by incorporation of a third acetyl-CoA molecule to form 3-hydroxy-3-methylglutaryl-CoA (HMG-CoA) via hydroxymethylglutaryl-CoA synthase (HmgB, HVO_2419). HMG-CoA is subsequently reduced to mevalonate by 3-hydroxy-3-methylglutaryl-coenzyme A reductase (HmgA, HVO_2583). Mevalonate is phosphorylated by mevalonate kinase (Mvk, HVO_2761) to yield mevalonate-5-phosphate, which is converted to isopentenyl-5-phosphate by phosphomevalonate decarboxylase (MvaD, HVO_1412). Isopentenyl phosphate kinase (IpK, HVO_2762) generates isopentenyl diphosphate (IPP), which is reversibly isomerized to dimethylallyl diphosphate (DMAPP) by isopentenyl diphosphate isomerase (IdiA, HVO_2506). Sequential prenyl chain elongation reactions catalyzed by bifunctional short chain isoprenyl diphosphate synthase (IdsA2, HVO_0303) produce geranyl diphosphate (GPP), farnesyl diphosphate (FPP), and geranylgeranyl diphosphate (GGPP). Phytoene synthase (CrtB, HVO_2524) converts two molecules of GGPP to phytoene, which is localized in the cell membrane and desaturated by carotenoid 3,4-desaturase (CrtD, HVO_2528) to yield lycopene (Brausemann et al., 2017; Palanisamy and Ramalingam, 2024). Lycopene is further modified by lycopene elongase/hydratase (Lye, HVO_2527) and bisanhydrobacterioruberin hydratase (CruF, HVO_2526), resulting in the production of the C_50_ carotenoid bacterioruberin. NADPH, nicotinamide adenine dinucleotide phosphate; ATP, adenosine triphosphate.

Halophilic archaea synthesize bacterioruberin through an NADPH-dependent mevalonate pathway (Miziorko, 2011; Zuo et al., 2018). Lycopene is a C_40_ precursor in this pathway and, like bacterioruberin, features an extensive conjugated double bond structure that contributes to its strong antioxidant properties (Tvrdá et al., 2016). In *Haloferax mediterranei*, the synthesis of lycopene requires *crtI* (HFX_2550) (Lin et al., 2021; Zuo et al., 2018), a phytoene desaturase gene homolog related to *H. volcanii crtD* (HVO_2528, carotenoid 3,4-desaturase) (Hartman et al., 2010), both belonging to arCOG01521. Figure 1 illustrates the proposed biosynthetic pathway for mevalonate and bacterioruberin synthesis in *H. volcanii*.

Beyond industrial relevance, carotenoids play critical roles in adaptation of microorganisms to extreme environments. Halophilic C_50_ carotenoids exhibit stronger antioxidant capacity and radical-scavenging activity than the C_40_ β-carotene (Hou and Cui, 2018; Baeza-Morales et al., 2025; Caimi et al., 2022; Lizama et al., 2021; Noby et al., 2023), thereby providing greater protection from UV-induced DNA damage and reactive oxygen species (Asgarani et al., 1999; Giani and Martínez-Espinosa, 2020; Hwang et al., 2024; Krinsky, 1993). Oxidative stressors such as H_2_O_2_ induce carotenoid accumulation in organisms such as *H. mediterranei* (Giani and Martínez-Espinosa, 2020), and these pigments display concentration-dependent antioxidant or pro-oxidant effects (Abbes et al., 2013; Ben Hamad Bouhamed et al., 2024). In addition, carotenoids enhance membrane rigidity and reduce water permeability, contributing to cellular stability under hypersaline conditions (Lazrak et al., 1988).

To meet growing industrial demand while reducing reliance on synthetic carotenoids (Heo et al., 2013; Li et al., 2019; Llorente et al., 2020; Stout et al., 2020), sustainable microbial approaches for carotenoid production are increasingly pursued. The model halophilic archaea, *Haloferax volcanii*, has emerged as a promising candidate for the sustainable production of value-added biochemicals (Giani et al., 2020; Kasirajan and Maupin-Furlow, 2021). Halophilic microorganisms have demonstrated metabolic versatility, including growth on low-cost industrial waste carbon sources such as candy starch and crude glycerin (Giani et al., 2024; Neagu et al., 2019). Crude glycerin, which accounts for approximately 10% (w/w) of biodiesel production during triglyceride transesterification, contains impurities such as methanol, water, salts, soaps, and non-glycerol organics, making its purification both costly and environmentally burdensome (Abdul Raman et al., 2019; Widayat and Hadiyanto, 2013; Yang et al., 2012; Zhang et al., 2022). The robust growth, reduced contamination risk, and genetic stability of *H. volcanii* make it particularly attractive for industrial bioprocessing (Haque et al., 2020; Hartman et al., 2010; Kasirajan and Maupin-Furlow, 2021; Oren, 2020). Collectively, these attributes position halophilic archaea, such as *H. volcanii*, as strong candidates for sustainable carotenoid biosynthesis through the valorization of industrial waste streams like crude glycerin.

Given the metabolic robustness and genetic accessibility of *H. volcanii*, this organism represents an ideal model system for enhancing bacterioruberin biosynthesis under industrial waste conditions (Haque et al., 2020; Hartman et al., 2010; Kasirajan and Maupin-Furlow, 2021). In this study, we found *H. volcanii* to exhibit high growth rate, biomass yield, and carotenoid production when cultured in minimal medium containing crude glycerin and urea as the sole carbon and nitrogen sources, compared to cultures supplemented with glycerol and ammonium chloride. Moreover, the regulatory mechanisms governing carotenoid 3,4-desaturase (*crtD*) expression remain largely unknown, as carotenoid biosynthesis is widely associated with cellular stress responses. Notably, we observe that urea supplementation enhances carotenoid production, and consistent with this, the *crtD* gene was found to be upregulated at the transcriptional level in crude glycerin-grown cells when urea served as the nitrogen source instead of ammonium chloride, suggesting a potential link between nitrogen-associated stress and pathway activation. By coupling the industrial waste stream of crude glycerin with the targeted optimization of the biosynthesis and regulation of carotenoids based on nitrogen source, this study advances sustainable strategies for improving carotenoid yields in halophilic production systems for a circular economy.

## Materials and methods

### Materials

Biochemical reagents were obtained from Fisher Scientific (Atlanta, GA, United States), Bio-Rad (Hercules, CA, United States), and Sigma-Aldrich (St. Louis, MO, United States). Oligonucleotide synthesis and DNA Sanger sequencing services were provided by Eurofins Genomics (Louisville, KY, United States). RNA extraction kit was purchased from Zymo Research (Irvine, CA, United States) and 2x RNA dye was purchased from Fisher Scientific (Atlanta, GA, United States). Luna qRT-PCR, Phusion High-Fidelity DNA Polymerase, restriction enzymes, DpnI, KLD enzyme mix, and 2x Quick Ligase were purchased New England Biolabs (NEB) (Ipswich, MA, United States). DNA fragments were isolated using NEB Monarch PCR and DNA Cleanup Kit or DNA Gel Extraction Kit (Ipswich, MA, United States). Pure glycerol of molecular biology grade was obtained from UltraPure MP Biomolecules (Irvine, CA, United States). Crude glycerin was from Lola Vega (Hollywood, FL, United States).

### Medium composition

Minimal medium (Hv-Min) was composed per liter of 144 g NaCl, 18 g MgCl_2_·6H_2_O, 21 g MgSO_4_·7H_2_O, 4.2 g KCl, 441 mg CaCl_2_·2H_2_O, 0.36 mg MnCl_2_·4H_2_O, 0.44 mg ZnSO_4_·7H_2_O, 2.3 mg FeSO_4_·7H_2_O, 0.05 mg CuSO_4_·5H_2_O, 51 mg uracil, 0.8 mg biotin, 0.1 mg thiamine, 10 mM NH_4_Cl or 10 mM urea, 9.75 mL of 0.1 M KPO_4_ buffer (pH 7.5), 0.18% (v/v) glycerol or crude glycerin, and 42 mL of 1 M Tris-Cl (pH 7.5) buffer. The glycerin/urea-mineral–vitamin supplement added to enrich basal medium was composed per liter of 441 mg CaCl_2_·2H_2_O, 0.36 mg MnCl_2_·4H_2_O, 0.44 mg ZnSO_4_·7H_2_O, 2.3 mg FeSO_4_·7H_2_O, 0.05 mg CuSO_4_·5H_2_O, 51 mg uracil, 0.8 mg biotin, 0.1 mg thiamine, 10 mM NH_4_Cl or 10 mM urea, 9.75 mL of 0.1 M KPO_4_ buffer (pH 7.5), and 0.18% (v/v) untreated crude glycerin. Crude glycerin concentration and composition was based on the Quality Trait Analysis report from Eurofins via mid and near infrared spectroscopy (Eurofins Genomics, Louisville, KY, United States): 87% glycerol, 4.2% water, 4.5% ash, and 1.3% methanol. Crude glycerin was diluted to a final concentration of 18.4% (v/v) using nanopure water and was neither filtered nor autoclaved; this preparation is hereafter referred to as crude glycerin. When crude glycerin was used as the carbon source in Hv-MM, the final concentrations of the crude glycerin components were: 0.184% glycerin, 0.0088% water, 0.0095% ash, 0.0027% methanol. ATCC974 medium, pH 6.8, was composed per liter of 125 g NaCl, 50 g MgCl_2_·6H_2_O, 5 g K_2_SO_4_, 0.134 g CaCl_2_·2H_2_O, 5 g tryptone, and 5 g yeast extract. LB medium was composed per liter of 10 g of NaCl, 10 g of tryptone, and 5 g of yeast extract and was supplemented with ampicillin (100 μg/mL) as needed. All agar plates were prepared by supplementing the medium with 15 g of agar per liter (1.5% w/v).

### General culture conditions

Strains and plasmids used in this study are listed in Table 1. Strains originated from a single isolated colony and were stored at −80 °C in 20% (v/v) glycerol stocks. *E. coli* strains were grown at 37 °C in LB medium with ampicillin included to select for plasmid constructs. *H. volcanii* strains were grown at 42 °C in Hv-MM supplemented with carbon and nitrogen sources as indicated. Small scale cultures (5 mL) were aerated in loosely capped culture tubes (13 × 100 mm) using a mini-rotator set at an ∼30° angle and 50–60 rpm (Glas-Col, Terre Haute, Indiana, United States; cat. no. 099A MR1512). Cultures grown in flasks were aerated by orbital shaking (200 rpm) using 50 mL cultures in 125 mL Erlenmeyer flasks (Pyrex No. 4980) and 100 mL cultures in 500 mL Erlenmeyer flasks (Pyrex No. 4980).

**TABLE 1:** List of *H. volcanii* strains and plasmids used in this study.

Name	Description	Ref.
*E. coli* strain		
TOP10	*F– recA1 endA1 hsdR17(r*_*K*_ *–m*_*K*_ *+) supE44 thi-1 gyrA relA1*	Invitrogen
GM2163	*F– ara-14 leuB6 fhuA31 lacY1 tsx78 glnV44 galK2 galT22 mcrA dcm-6 hisG4 rfbD1rpsL136 dam13::Tn9 xylA5 mtl-1 thi-1 mcrB1 hsdR2*	New England Biolabs
*H. volcanii* strain		
DS2	Dead Sea isolate	Mullakhanbhai and Larsen (1975)
DS70	DS2 cured of plasmid pHV2	Wendoloski et al. (2001)
H26	DS70 *ΔpyrE2*	Allers et al. (2004)
Plasmid		
pJAM2678	Amp^R^, nov^R^; pJAM202-derived plasmid containing P2_*rrnA*_-*bgaH* from pTA102	Rawls et al. (2010)
pJAM2714	Amp^R^, nov^R^; pJAM2678-derived plasmid devoid of P2_*rrnA*_ from pTA102 and Shine Dalgarno sequence	Rawls et al. (2010)
pJAM2715	Amp^R^, nov^R^; pJAM2678-derived plasmid devoid of P2_*rrnA*_ from pTA102	Rawls et al. (2010)
pJAM4483	Amp^R^, nov^R^; pJAM2678 containing P_*HVO_2528*–237 bp*-*_ *bgaH*	This study

### Isolated colonies

All *H. volcanii* assays were initiated from single colonies that were isolated by quadrant streaks, using a toothpick for the initial quadrant and a wire loop for the remainder of the streaks. Strains were inoculated from 20% (v/v) glycerol stocks (−80 °C) onto glycerol minimal medium (Hv-GMM) 1.5% (w/v) agar plates supplemented with a nitrogen source of either 10 mM ammonium chloride (NH_4_Cl) or 10 mM urea as indicated. Pure glycerol (0.18% laboratory grade glycerol), rather than crude glycerin, was used the agar plate formulation; otherwise, the medium composition was identical to that used for liquid cultures in downstream analyses, as indicated. Plates were incubated at 42 °C until isolated colonies were observed (4–5 days).

### Growth assays

To determine growth metrics, single colonies were cultured with 5 mL Hv-MM supplemented with a mix of 0.18% (v/v) glycerol or crude glycerin and 10 mM NH_4_Cl or urea in rotating culture tubes (13 × 100 mm). All cells were grown to log phase (OD_600_ 0.5–0.6) and sub-cultured to OD_600_ 0.02 with 5 mL respective medium and grown again to log phase. Cells were sub-cultured again to OD_600_ 0.02 into 1 mL respective medium in 1.5 mL Eppendorf tubes and briefly (5–10 min) incubated with rotation at 42 °C prior to aliquoting. In a 96-well CellPro cell culture plate (Alkali Scientific, FL), 150 µL of sub-culture was aliquoted into replicate wells. Using the Synergy Epoch 2 microplate reader and Gen5 software (Agilent, Santa Clara, CA), cell growth was measured as follows: OD_600_ was measured every 15 min for 99 h, with aeration (orbital continuous shaking), and temperature setpoint 42 °C. No inoculum controls were included to assess potential background signals from the medium alone.

### Cultivation for carotenoid extraction based on glycerol and nitrogen source

Single colonies were inoculated into 5 mL of Hv-minimal medium (Hv-MM) supplemented with combinations of either 0.18% glycerol or 0.18% crude glycerin and 10 mM NH_4_Cl or 10 mM urea, in 13 × 100 mm culture tubes and incubated with aeration at 42 °C. Cells were grown to log phase (OD_600_ of 0.6). Cells were then sub-cultured to an OD_600_ 0.01 in 100 mL of the respective media in 500 mL flasks and grown to stationary phase (OD_600_ of 0.8 for NH_4_Cl and 1.2 for urea) with aeration. All cultures were harvested by centrifugation for carotenoid extraction as detailed below.

### Reuse of spent medium

For reuse of spent medium, single colonies were inoculated into 5 mL of Hv-minimal medium (Hv-MM) supplemented with 0.18% crude glycerin and 10 mM urea, in 13 × 100 mm culture tubes and incubated at 42 °C with rotary shaking at 200 rpm until cells reached log phase (OD_600_ of 0.6). Cells were then sub-cultured to an OD_600_ 0.01 in 100 mL of the respective media in 500 mL flasks and grown to stationary phase (OD_600_ of 1.2) for 3 days at 42 °C (200 rpm). Cultures (100 mL) were harvested (4,000 x *g*, 30 min, 4 °C). The supernatant was collected into Pyrex 1L round media storage bottles and was considered “basal medium and inoculum”, as this spent medium contained the salts and residual cells that carried over from the previous culture. The spent medium (91.13 mL), which included a carryover of basal salts and cells, was mixed with the glycerin/urea-mineral–vitamin supplement (8.86 mL). No additional cells were used to inoculate the culture medium. The cultures (100 mL total) were incubated (42 °C, 200 rpm) in 500 mL flasks for 2–3 days until cells reached stationary phase (OD_600_ of 1.2). The reuse of spent medium was repeated for a total of 3 times, therefore having one starter culture and 3 re-use cultures. All cultures were harvested by centrifugation for carotenoid extraction as detailed below.

### Glycerol or crude glycerin supplementation at stationary phase for continued cultivation

For the supplementation of additional glycerol or crude glycerin, single colonies were inoculated into 5 mL of Hv-minimal medium (Hv-MM) supplemented with 10 mM urea and either 0.18% crude glycerin or glycerol, in 13 × 100 mm culture tubes. Cultures were incubated at 42 °C with rotary shaking at 200 rpm until cells reached log phase (OD_600_ of 0.6). Cells were then sub-cultured to an OD_600_ 0.01 in 100 mL of the respective media (in 500 mL flasks) and grown for 2–3 days until cells reached stationary phase (OD_600_ of 1.0–1.2). The stationary phase cultures were then supplemented with an addition of 0.18% glycerol or crude glycerin and grown an additional 2 days. A control flask of cells grown without addition of 0.18% glycerol was also grown for 2 additional days. The final OD_600_ was measured. A portion of the culture (1 mL) was harvested by centrifugation (4,000 x *g*, 30 min, 4 °C) and used to determine protein concentration per mL culture. The rest of the culture (∼100 mL) was harvested by centrifugation for carotenoid extraction as detailed below.

### Large scale cultivation

For large scale production of carotenoids, single colonies were inoculated into 100 mL of Hv-minimal medium (Hv-MM) supplemented with 10 mM urea and 0.18% crude glycerin in a 500 mL flasks (no. 4890). Cultures were incubated at 42 °C with rotary shaking at 200 rpm until cells reached log phase (OD_600_ of 0.6). Cells were then sub-cultured to an OD_600_ 0.01 in 500 mL of the respective media in 2.8 L flasks (no. 4420) for a total of 4 L. Cells were grown for 3 days until cells reached stationary phase (OD_600_ of 1.0–1.2). The supernatant was collected into Pyrex 1L round media storage bottles and was considered “basal medium and inoculum”. The spent medium (455.65 mL), which included a carryover of basal salts and cells, was mixed with the glycerin/urea-mineral–vitamin supplement (44.3 mL). No additional cells were used to inoculate the culture medium. The cultures (500 mL total) were incubated (42 °C, 200 rpm) in 2.8 L flasks (no. 4420) flasks for 3 days until cells reached stationary phase (OD_600_ of 1.2). The stationary phase cultures were then supplemented with an addition of 0.18% crude glycerin and grown an additional 2 days. The cultures (500 mL x 8) was harvested by centrifugation for carotenoid extraction as detailed below.

### Determining mg protein per mL culture

Cell pellets (derived from 1 mL aliquot of 100 mL culture) were lysed in 500 μL 20 mM HEPES [pH 7.5] and 2 M NaCl by sonication using a Branson Sonifier Cell Disruptor 200. Sonication was performed in 30 s cycles, followed by 1-min intervals on ice, for a total of 3 cycles. The high salt buffer was used to ensure protein stability. Protein concentration was quantified using the Quick Start Bradford 1× Dye Reagent (Bio-Rad cat. no. 5000205) or according to the manufacturer’s instructions. Samples (5 µL) were placed in a 96-well plate in triplicate. A standard curve was prepared by diluting BSA (bovine serum albumin) standards in the lysis buffer across a range of concentrations. Protein concentration (mg·mL^-1^) is calculated as mg of protein per mL of cell culture.

### Carotenoid extraction and analysis

All cultures were grown in the dark and tubes were covered with tin foil to minimize light exposure. Cultures (100 mL) were harvested by centrifugation (4,000 x *g*, 30 min, 4 °C) with a swing bucket rotor (ThermoFisher TX-1000) and used immediately for carotenoid extraction. During method optimization, carotenoid extraction from frozen cell pellets (−80 °C) using 100% acetone led to cellular dehydration and reduced pigment recovery. Therefore, extractions were subsequently performed using freshly harvested cells. Carotenoid extraction was performed as previously described with the following with modifications (Churio et al., 2022; Zalazar et al., 2019). For 100 mL cell culture, 5 mL of 100% acetone was added, and the suspension was subjected to sonication for 20–30 s to disrupt the cells. Although sonication was not required for routine handling, brief sonication was preferred compared to pipetting for carotenoid recovery. The resulting cell-acetone mixture was incubated overnight at 4 °C to facilitate carotenoid extraction on a rocking platform (Lab-Line Maxi Rotator). Following incubation, the samples were centrifuged (4,000 × g, 15 min, 4 °C) to separate the cellular debris from the acetone-soluble carotenoids. The supernatant containing the carotenoids was transferred to 15 mL Falcon tube and stored at −20 °C. The remaining cell pellet was resuspended in 1–2 mL of 100% acetone, and the extraction procedure was repeated to ensure complete removal of carotenoids, as indicated by a visibly white pellet. This process was repeated if needed. For determining spectral profile, UV-visible spectroscopy analysis was used. Carotenoid samples (1 mL) were loaded into a quartz cuvette (Quartz Spectrophotometer cell Micro 16.50-Q-10/8.5 mm, Bio-Rad) and spectral scans were recorded from 350 to 650 nm (40 Vis/UV-Vis Spectrophotometers, Genesy, ThermoFisher).

Carotenoid concentration (mg·L^-1^) was determined based on the molar extinction coefficient at A492 nm of 185,288 M^-1^cm^-1^ and molecular mass of 741.15 g/mol of bacterioruberin (Biswas et al., 2016; Churio et al., 2022). Carotenoid concentrations were first calculated using Beer–Lambert law to obtain mg·L^-1^ values in the acetone extract. These values were then multiplied by the final acetone extraction volume (Supplementary Table S1), thus reporting the value mg·L^-1^ of carotenoid recovered from the 100 mL culture. All values presented in Figure 3 are reported using this calculation. The final extract volume was determined by carefully collecting the upper acetone layer following separation from the cellular debris. To note, occasionally a higher density yellow layer will form with media carry over. Carotenoid yield was additionally normalized to the lowest OD_600_ value to account for differences in culture density. For each sample, a normalization factor was calculated by dividing its OD_600_ by the minimum OD_600_ observed across all conditions for that test. The measured carotenoid yield was then divided by this normalization factor to obtain the OD_600_-normalized yield.

For Table 4, the total carotenoid recovered in the acetone extract was normalized to the culture volume and expressed as mg·L^-1^ of cell culture. This represents the only case in which pigment concentrations are presented on a per-liter-of-culture basis.

### RNA extraction and analysis

For nitrogen source dependent analysis, single colonies were inoculated into 5 mL Hv-minimal medium (HvMM) supplemented with glycerol and 10 mM ammonium chloride (NH_4_Cl) or urea as the nitrogen source and grown in culture tubes (13 × 100 mm) at 42 °C with aeration. Cultures were sub-cultured to an optical density at 600 nm (OD_600_) of 0.02 in 5 mL of the respective medium in 13 × 100 mm culture tubes and incubated under the same conditions. A third subculture was made to an optical density at 600 nm (OD_600_) of 0.02 in 5 mL of the respective medium in 13 × 100 mm culture tubes incubated under the same conditions. Once cells reached an OD_600_ of 0.4–0.6 (log phase) and 0.8–1.2 (stationary phase), 1 mL of each culture was harvested by centrifugation at 5,000 × g for 10 min at room temperature (RT). For NH_4_Cl grown cells, log and stationary phase occurred at a lower OD_600_ then cells grown in urea. As a control, single colonies were inoculated into 5 mL ATCC974 rich medium grown in culture tubes (13 × 100 mm) and incubated at 42 °C with aeration with the same protocol. RNA extraction was performed using the Quick-RNA MiniPrep Plus Kit (cat. no. R1057, Zymo) according to the manufacturer’s protocol. RNA integrity was assessed via electrophoresis on a 0.8% (w/v) agarose gel containing 2× RNA loading dye (cat. no. B0363S, NEB). Samples were diluted to a final concentration of 0.4 ng·μL^-1^ and stored at −80 °C until further use.

### qRT-PCR analysis

Primers used for qRT-PCR analyses are listed in Table 2. The Luna Universal One-Step RT-qPCR Kit (cat. no. E3005, NEB) was utilized for qRT-PCR analysis using the CFX96 Real-Time C1000 Thermal Cycler (Bio-Rad). The reverse transcription reaction was carried out at 55 °C for 10 min. The qRT-PCR cycling parameters consisted of an initial denaturation at 95 °C for 1 min, followed by 40 cycles of 95 °C for 10 s and 56 °C for 30 s (plate read), concluding with a final extension at 60 °C for 30 s. A melting curve analysis was conducted by denaturing at 95 °C for 10 s, then gradually increasing the temperature from 60 °C to 95 °C in 5-s increments. A single peak in the melt curve confirmed the specificity of the amplification. Gene expression of the carotenoid 3,4-desaturase *crtD* (HVO_2528) was normalized using the housekeeping gene encoding the large ribosomal subunit protein (RpL-16, HVO_0484). The 2^−ΔΔCT^ method was employed to determine relative changes in mRNA levels. Statistical differences in gene expression between the H26 parental strain and various medium conditions were evaluated using Student’s t-test. Primer efficiency was validated with genomic DNA as the template, and only primer pairs with efficiencies ranging from 95% to 105% and an *R* ^2^ value between 0.9 and 1.10 were included in the analysis. A qRT-PCR control lacking the reverse transcriptase step was included to rule out genomic contamination of the RNA preparations.

**TABLE 2:** List of *H. volcanii* primers used in this study.

Name	Sequence (5′ to 3′)	Ref.
p.*crtD* Xbal^a^	AATTCT​AGAGGA​TAT​CGT​TGA​CGA​CCG​ACT​G	This study
p.*crtD* promoter NdeI^a^	TTGCAT​ATGGTC​CGT​TCG​TAA​GGA​CGT​AGA​G	This study
*crtD* F qRT-PCR^b^	CCC​GAA​GCT​CCA​GCA​GAT​AA	This study
*crtD* R qRT-PCR^b^	TTG​AAG​TCG​ACG​TGG​CTC​AT	This study
RpL-16 (HVO_0484) F qRT-PCR^b^	GCG​AGT​ACA​TCA​CGG​GTA​TC	Mondragon et al. (2022)
RpL-16 (HVO_0484) R qRT-PCR^b^	CAC​TTC​CTC​TTC​GAC​CTT​CAG	Mondragon et al. (2022)

^a^ Used for construction of plasmid pJAM4483.

^b^ Used for qRT-PCR.

### Beta-galactosidase strain generation

Primers used for plasmid construction are listed in Table 2. The *Haloferax alicantei bgaH*, encoding β-galactosidase, was derived from pTA102 and was cloned into pJAM202 to generate pJAM2678. The promoter region (237 bp) upstream of the *crtD* start codon was amplified from *H*. *volcanii* H26 genomic DNA extracted by the spooling method (Tang et al., 2009). The promoter region of *crtD* was fused to *bgaH* of pJAM2678 to generate pJAM4483 with restriction enzyme digestion (Xbal and NdeI) and ligation. Plasmids were transformed sequentially into *Escherichia coli* TOP10, *E. coli* GM2163, and *H. volcanii* H26. Controls included pJAM2678 without a gene promoter, pJAM2714 with the removal of the *H. cutirubrum* P2_*rrnA*_ promoter and the Shine Dalgarno sequence, and pJAM2715 removal of only the P2_*rrnA*_ promoter. *H. volcanii* H26 was transformed using a PEG-based method developed by Cline et al. (Cline et al., 1989) as outlined in the *Halohandbook* (Tang et al., 2009).

### Beta-galactosidase activity assay

To evaluate promoter activity, a β-galactosidase (*bgaH*) reporter assay was conducted using 5-bromo-4-chloro-3-indolyl β-D-galactopyranoside (x-gal)-based colorimetric screening and o-nitrophenyl- β-D-galactopyranoside (ONPG)-based enzymatic quantification. *H. volcanii* strains were inoculated from 20% (v/v) glycerol stocks (−80 °C) onto Hv-minimal medium (HvMM) supplemented with glycerol, either 10 mM ammonium chloride (NH_4_Cl) or urea as the nitrogen source or ATCC974 rich medium, and 1.5% (w/v) agar. Plates were incubated at 42 °C for 5 days. For detection of β-galactosidase activity, plates were sprayed with x-gal prepared at a concentration of 10 mg·mL^-1^ in dimethylformamide. For color development, the sprayed plates were incubated at 37 °C for 24 h. Colonies exhibiting active promoter-driven β-galactosidase expression developed a blue coloration resulting from β-galactosidase-mediated hydrolysis of X-gal producing a highly concentrated, insoluble indigo dye that visually overrides the native red pigmentation due to bacterioruberin.

For quantitative analysis, single colonies were inoculated into 5 mL of corresponding HvMM or ATCC974 rich liquid media in 13 × 100 mm culture tubes and incubated at 42 °C with aeration. Cells were grown to exponential phase (OD_600_ of 0.6) and then sub-cultured in 50 mL respective media to an OD_600_ of 0.02 and grown until OD_600_ of 0.3–0.4 for NH_4_Cl and OD_600_ of 0.5–0.6 for urea. An aliquot (5 mL) of the culture was harvested by centrifugation at 5,000 × *g* for 20 min in 15 mL falcon tubes, and the cell pellets were stored in −80 °C until further use. The same culture (∼45 mL) was further incubated under the same conditions until late log phase (OD_600_ of 0.7–0.8 for NH_4_Cl and OD_600_ of 1.0–1.2 for urea). An aliquot (5 mL) of the culture was harvested under the same conditions. Pellets were washed once in 1 mL of Buffer B (50 mM Tris-HCl (pH 7.2), 2.5 M NaCl, and 10 µM MnCl_2_), recentrifuged (5,000 × *g*, 20 min, 4 °C), and resuspended in 300 µL of *bgaH* lysis buffer (buffer B supplemented with 0.5% (vol/vol) β-mercaptoethanol). Cells were lysed by sonication for 3 × 10 s on/10 s off (with intermittent chilling on ice), and insoluble debris was removed by centrifugation (5,000 × *g*, 20 min, 4 °C). Protein concentration of the cell lysate was quantified using a Bradford assay following the manufacturer’s instructions (1× Quick Start Bradford Reagent), using BSA standards prepared in *bgaH* buffer. For the assay, 10 µg of total protein was added to *bgaH* buffer to a final reaction volume of 90 µL. Reactions were prepared in triplicate in a 96-well CellPro cell culture plate (Alkali Scientific, FL). The assay was initiated by adding 10 µL of fresh 10x ONPG stock solution (2.66 mM ONPG in 100 mM potassium phosphate buffer, pH 7.2), yielding a final volume of 100 µL per well. Final reactions (100 µL) were composed of 10 μg cell lysate and 0.266 mM ONPG in 50 mM Tris-HCl (pH 7.2), 2.5 M NaCl, and 10 µM MnCl_2_. Reactions were carried out at 25 °C and monitored using a BioTek Epoch 2 microplate reader with Gen5 software (Agilent, Santa Clara, CA). Prior to kinetic measurements, the 96-well plates were subjected to 10 s of continuous double-orbital shaking. β-galactosidase activity then was monitored at 25 °C every 1.5 min for 16 h be measuring an increase in absorbance 405 nm (A_405nm_). Activity was expressed in mU·mg^-1^ protein, where 1 Unit (U) is defined as the amount of enzyme that generates 1 µmol of ortho-nitrophenol (ONP) per min. Product formation was quantified spectrophotometrically using the molar extinction coefficient of ONP (ε = 3,300 M^-1^ cm^-1^). Negative controls (*i.e*., buffer, cell lysate, or buffer and OPNG) were included for each condition.

### Statistical analysis

All experiments were performed at least twice and included three independent biological repeats and three technical replicates. Results are reported as mean ± standard deviation (SD). Statistical differences were evaluated using Student’s t-test, with significance defined as p < 0.05 or p < 0.005 or non-significant (n.s.). Analyses were carried out in Microsoft Excel.

## Results

### Crude glycerin and urea promote high biomass and carotenoid pigmentation in *H. volcanii*

**FIGURE 2:**
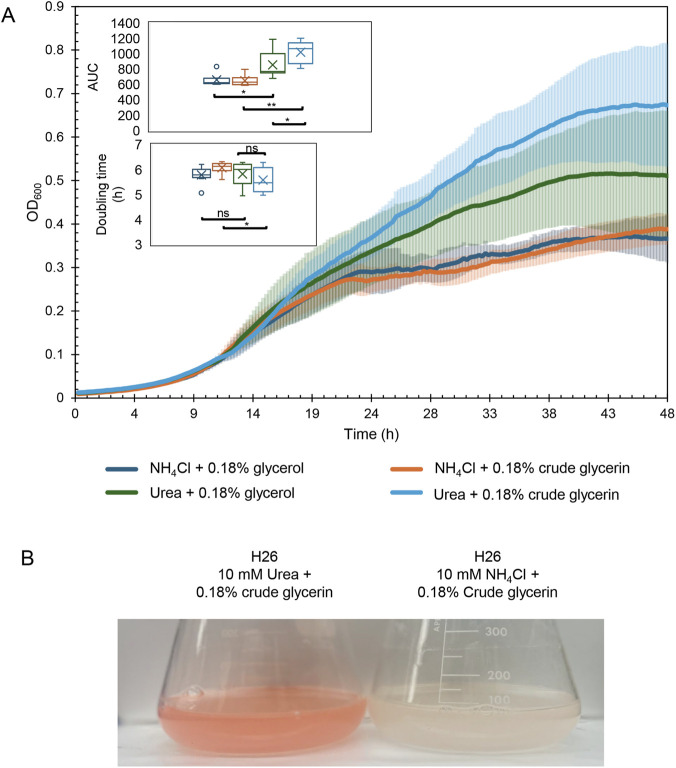
Growth of *H. volcanii* strain H26 using laboratory-grade glycerol (glycerol) or biodiesel waste (crude glycerin) and urea or ammonium chloride (NH_4_Cl) as sources of carbon and nitrogen. **(A)** Growth curves with insets comparing area under the curve (AUC) and doubling time values. *H. volcanii* H26 was grown at 42 °C in Hv-MM supplemented with 0.18 (v/v)% glycerol or crude glycerin and 10 mM NH_4_Cl or urea as indicated. Growth was monitored at OD_600_ in 96-well plates using a Synergy BioTek microtiter plate reader. Dark blue, glycerol and NH_4_Cl; orange, crude glycerin and NH_4_Cl; green, glycerol and urea; light blue, crude glycerin and urea. Data shown as mean ± SD (n = 3 biological replicates with 3 technical replicates). Statistical significance assessed using a Student’s t-test (p-value ≤0.005, **; ≤0.05, *; not significant, ns). Statistically significant differences in AUC among treatments: glycerol with NH_4_Cl verses urea (p-value 0.0056), crude glycerin with NH_4_Cl verses urea (p-value 1.56 × 10^−6^), and urea with glycerol verses crude glycerin (p-value 0.028). Doubling time among treatments: crude glycerin with NH_4_Cl verses urea (p-value 0.0069). **(B)** Deep pigmentation of H26 observed when optimized for growth in crude glycerin using urea verses NH_4_Cl the nitrogen source. Cultures (100 mL) were in 500 mL flasks, and images were captured at stationary phase.

The ability of *H. volcanii* to utilize crude glycerin as the sole carbon source and urea as a nitrogen source was evaluated by growth assays. Growth of strain H26 was assessed in Hv-MM supplemented with either 10 mM urea or NH_4_Cl as the nitrogen source and 0.18% (v/v) laboratory-grade glycerol (referred to as glycerol) or untreated crude glycerin derived from biodiesel waste as the carbon source. Growth was monitored over 48 h by OD_600_, enabling calculation of growth metrics including area under the curve (AUC), growth rate (h^-1^), doubling time (h) and maximum OD_600_ (Figure 2A; Table 3). A negative control containing medium with untreated crude glycerin and without H26 was included, and no growth was observed. While growth rates and doubling times were relatively similar among the various culture conditions, AUC and maximum OD_600_ values indicative of biomass yield were found to differ. When cells used NH_4_Cl as the sole nitrogen source, lower yields of biomass were observed on glycerin compared to glycerol. However, when urea served as the nitrogen source, crude glycerin supported higher biomass than glycerol. In general, cultures grown with urea achieved a greater maximum OD_600_ (0.5–0.6) compared to those grown with NH_4_Cl (0.38). Moreover, the urea-grown cultures exhibited deep pink pigmentation, in contrast to the off-white coloration observed in NH_4_Cl-grown cells (Figure 2B). Together these results reveal *H. volcanii* can efficiently metabolize crude glycerin derived from biodiesel waste, supporting comparable growth rates and higher biomass than that observed with laboratory-grade glycerol. In addition, urea was found to be important in enhancing this biomass accumulation, as indicated by maximum OD_600_, and appeared to promote carotenoid production, evidenced by the deep pink pigmentation of the cultures.

**TABLE 3:** Growth metrics of *H. volcanii* H26 on Hv-MM supplemented with the nitrogen-source of NH_4_Cl vs. urea and carbon-source of glycerol vs. crude glycerin^a^.

Hv-minimal medium (Hv-MM)	Cell growth parameters			​
Carbon and nitrogen source	AUC	Growth rate (h^-1^)	Doubling time (h)	Maximum OD_600_
0.18% glycerol +10 mM NH_4_Cl	660 ± 72.7	0.12 ± 0.01	5.79 ± 0.33	0.36
0.18% crude glycerin +10 mM NH_4_Cl	649 ± 66.3	0.11 ± 0.00	6.07 ± 0.22	0.38
0.18% glycerol +10 mM urea	855 ± 191	0.12 ± 0.01	5.83 ± 0.49	0.51
0.18% crude glycerin +10 mM urea	1,020 ± 145	0.12 ± 0.01	5.58 ± 0.49	0.67

^a^ AUC, area under the curve calculated from 0 to 48 h; growth rate and doubling time determined from 9 h to 24 h; maximum OD_600_ based on OD_600_ reached at 48 h when grown in a 96-well plate.

### Carotenoid biosynthesis in *H*. *volcanii* is stimulated under urea supplementation

**FIGURE 3:**
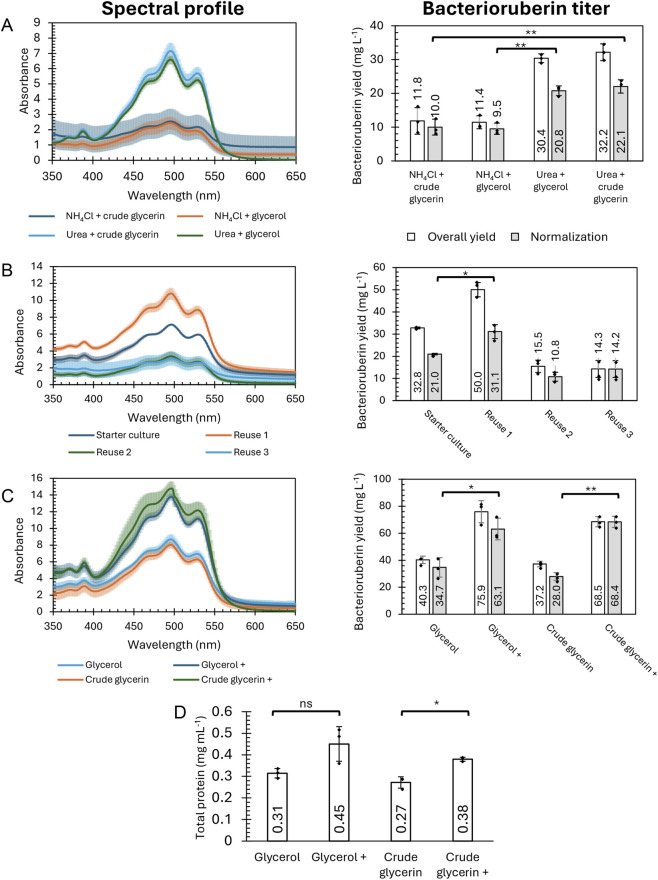
Bacterioruberin yield of *H. volcanii* H26 grown in Hv-MM supplemented with different carbon and nitrogen sources. Hv-MM supplemented with 10 mM NH_4_Cl or 10 mM urea and 0.18% (v/v) glycerol or 0.18% (v/v) crude glycerin was used unless otherwise indicated. **(A)** Carotenoid yield of cells cultivated in Hv-MM with different sources of nitrogen (NH_4_Cl or urea) and carbon (glycerol or crude glycerin). Dark blue, NH_4_Cl + crude glycerin; orange, NH_4_Cl + glycerol; Light blue, urea + crude glycerin; green, urea + glycerol. **(B)** Carotenoid yield of cells cultivated from spent broth of Hv-MM cultures containing urea and crude glycerin, followed by supplementation with a glycerin/urea–mineral–vitamin mix (3 times, re-use 1-3). Dark blue, initial ‘starter’ culture; orange, re-use 1; Light blue, re-use 2; green, re-use 3. **(C)** Carotenoid yield of cells cultivated in Hv-MM with urea and different carbon (glycerol or crude glycerin) sources and then replenished in stationary phase with glycerol or crude glycerin. Light blue, glycerol; dark blue, glycerol + additional glycerol; orange, crude glycerin; green, crude glycerin + additional crude glycerin. Left, Representative spectral profile of extracted carotenoid displayed as wavelength versus absorbance. Absorbance of the crude extract measured across the visible spectrum of 350–650 nm. Right, Bacterioruberin titer expressed as (mg·L^-1^ acetone) as indicated on y-axis. Carotenoid content was calculated based on the maximum absorption of bacterioruberin observed at 492 nm. Samples were analyzed as overall yield (white) or adjusted to the lowest OD_600_ value (gray). Data is shown as mean ± SD (n = 3 biological replicates). Carotenoid yields were normalized to the lowest OD_600_ value by scaling raw yields according to relative culture density. Statistical significance assessed using a Student’s t-test (p-value ≤0.005, **; p-value ≤0.05, *). Significant differences were observed between NH_4_Cl- and urea-grown cultures when glycerol was used as the carbon source (p-value 0.0009) and crude glycerin (p-value 0.002) (panel A, right). Upon reuse of cultures (reuse 1), the difference between the starter culture was significant (p-value 0.02) (panel B, right). Supplementation with an additional carbon source also resulted in significant changes in carotenoid yield. Glycerol-grown cultures supplemented with glycerol differed significantly from un-supplemented cultures (p-value 0.01) and crude glycerol grown cultures verses the addition of crude glycerin (p-value 0.0003) (panel C, right). **(D)** Total protein concentration (mg·mL^-1^ culture) from cultures on panel C were measured in triplicates to assess biomass. In Hv-MM supplemented with 10 mM urea and 0.18% (v/v) glycerol, total protein reached 0.31 ± 0.02 mg·mL^−1^, while urea with 0.18% (v/v) crude glycerin yielded 0.27 ± 0.02 mg·mL^−1^. Once cells reached stationary phase, additional 0.18% glycerol increased total protein to 0.45 ± 0.08 mg·mL^−1^, and additional 0.18% crude glycerin resulted in 0.38 ± 0.009 mg·mL^−1^. Statistical significance assessed using a Student’s t-test (p-value ≤0.05, *; not significant, n.s.). Differences between with and without additional glycerol conditions were statistically significant (p-value ns), as were differences between crude glycerin treatments (p-value 0.012). Further details regarding carotenoid yields associated with this figure are provided in Supplementary Table S2.

The visible differences in cell pigmentation observed among the cultures (Figure 2B) suggested carotenoid content may be stimulated by urea (vs. NH_4_Cl) when cells are grown on either crude glycerin or glycerol. To quantify this effect, carotenoids were extracted from cells grown under defined culture conditions, and the carotenoid content was determined spectrophotometrically. UV-visible absorption spectra of the extracted carotenoids were recorded over the visible range of 350–650 nm (Figure 3, left). The spectra were found to exhibit absorption maxima at approximately 372, 388, 470, 496, and 530 nm, consistent with the C_50_ carotenoid bacterioruberin (Abbes et al., 2013; Ben Hamad Bouhamed et al., 2024; Churio et al., 2022; Verma et al., 2020). Thus, the spectral properties of bacterioruberin and its molecular mass were used to calculate the carotenoid yield (Figure 3, right).

Using this approach, cultures supplemented with urea were found to produce higher carotenoid concentrations than those supplemented with NH_4_Cl, independent of the carbon source (either glycerol or crude glycerin) (Figure 3A). Unless otherwise indicated, pigment concentrations are reported in mg·L^-1^ based on 1 mL of acetone extract derived from 100 mL of cell culture. After normalization, the combination of urea and crude glycerin resulted in the highest pigment concentration (22.1 ± 2.0 mg·L^-1^). This was followed by cultures supplemented with urea and glycerol, which yielded 20.8 ± 1.4 mg·L^-1^. In contrast, cultures grown with NH_4_Cl and glycerol or crude glycerin exhibited approximately threefold lower yields (9.5 ± 1.7 mg·L^-1^ and 10.0 ± 2.4 mg·L^-1^, respectively). When normalized to the lowest OD_600_ value and compared to urea, growth using NH_4_Cl as a nitrogen source reduced carotenoid production by 2.3-fold in glycerol-grown cells and 2.1-fold in crude glycerin-grown cells. Overall, urea was the superior nitrogen source, when compared to NH_4_Cl, for carotenoid production from crude glycerin.

### Reuse of spent medium

As high concentrations of salt remain in the medium after cell harvesting, the spent medium was reused to evaluate its potential for additional cultivation cycles (Figure 3B). Spent medium was considered the “basal medium and inoculum” and a glycerin/urea-mineral–vitamin supplement was used to replenish nutrients during every re-use cycle. After normalization, carotenoid production was found highest after the first reuse (31.1 ± 3.1 mg·L^-1^), with 1.5-fold higher values than the initial culture (21.0 ± 0.3 mg·L^-1^). However, each subsequent reuse was found to reduce pigment content (10.8 ± 2.0 and 14.2 ± 3.8 mg·L^-1^) in comparison to the initial and first reuse cultures. Thus, a single reuse of spent medium appears useful for not only reducing nutrient medium costs and eliminating the need for fresh cellular inoculum but also enhancing carotenoid yield.

### Cell density increases with supplemental crude glycerin in stationary cultures

As *H. volcanii* readily metabolized crude glycerin as the carbon source, we tested whether additional doses of glycerol or crude glycerin at stationary phase could further enhance cellular biomass and/or carotenoid yield. Thus, cells were grown to stationary phase with 10 mM urea and either 0.18% (v/v) glycerol or crude glycerin, provided with a second supplement of the respective carbon source, and then incubated for an additional 2 days. Un-supplemented cultures were incubated for an equivalent duration. Cells harvested with or without stationary-phase supplementation were subsequently compared for carotenoid concentration (Figure 3C) and total protein content (Figure 3D), the latter a readout of biomass. After normalization and comparison to levels prior to additional carbon supplementation, cultures receiving extra glycerol or crude glycerin at stationary phase exhibited 1.8-fold and 2.4-fold increases in carotenoid yield, respectively (Figure 3C). Moreover, total protein concentration (mg·mL^-1^ culture) was found to increase 1.4-fold after stationary-phase supplementation with either type of glycerol source (Figure 3D). Total protein content was used to measure biomass rather than OD_600_ due to limitations in optical density measurements for *H. volcanii*, where carotenoid pigmentation and variability in cell morphology can interfere with optical density (OD_600_) readings. Accordingly, total protein provides a reliable and quantitative measure of biomass under these conditions. Overall, these results reveal that the stationary phase cells have retained the capacity to convert added glycerol or crude glycerin to carotenoids and cellular biomass under these culture conditions.

### Optimization of cell density and carotenoid yield

To further optimize carotenoid yield, the strategies of using the spent medium (reuse 1) and supplementing stationary-phase cells with an additional dose of 0.18% crude glycerin were combined. These cultures were found to reach an OD_600_ of 2.33 ± 0.17 and total protein concentration of 0.47 ± 0.03 mg·mL^-1^ culture. Carotenoids were extracted from the 100 mL cultures. Based on a 1 mL acetone extract, the carotenoid yield was 90.4 ± 4.8 mg·L^-1^. When normalized to the lowest OD_600_ value, the yield was 84.1 ± 8.4 mg·L^-1^. Carotenoids were extracted from 4.0 L cultures grown under medium reuse and additional stationary-phase crude glycerin supplementation (0.18% per addition). Under these conditions, carotenoid yields reached 1,698 mg·L^-1^ acetone extract, corresponding to 0.425 mg·L^-1^ of culture. Further investigation is required to refine cultivation conditions and develop scalable processes for enhanced carotenoid production. Overall, this data indicates that combining the optimized reuse and glycerin supplementation strategies supports high-density growth and efficient carotenoid biosynthesis.

### Increased transcript abundance of the carotenoid biosynthetic gene *crtD* during growth on urea

**FIGURE 4:**
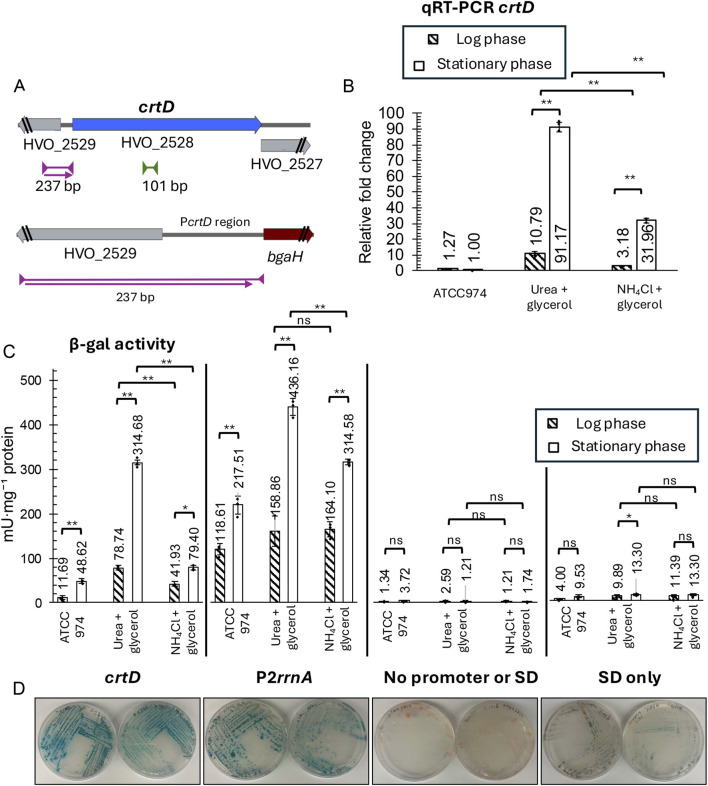
*H. volcanii* carotenoid 3,4-desaturase gene (*crtD*) and its transcriptional regulation based on nitrogen source. **(A)** Schematic representation of *crtD* with the location of the primers used in qRT-PCR and the generation of the β-galactosidase (β-gal) reporter indicated (Top). Represented: Blue, *crtD*. Grey, neighboring genes. Purple, primers used to amplify a 237 bp region and fuse the *crtD* promotor region to the β-galactosidase gene (*bgaH*) of *H. alicantei*, enabling β-galactosidase based detection of promoter activity. Purple arrow, orientation of promoter-reporter fusion construct. The green arrows represent qRT-PCR primer binding locations. Purple arrows denotes the orientation of the *crtD*–*bgaH* fusion construct. Bottom represents the zoomed in 237 bp *crtD*–*bgaH* fusion construct. Grey, neighboring gene. Burgundy, *bgaH* from *H. alicantei*. P*crtD* region outlined. The purple blue arrow represents the transcriptional orientation of the promoter–reporter fusion construct. Black lines indicate that the gene extends beyond the region shown. Primer DNA sequences are listed in Table 2. **(B)** Transcript abundance of *crtD* as determined by qRT-PCR. Average relative fold change of transcript levels for *crtD* under varying nitrogen sources at log and stationary phase via qRT-PCR determined by the ΔΔCt method. Transcript levels were normalized against the internal reference *rpL-16* gene. Data shown as mean ± SD (n = 3 biological replicates and 3 technical replicates). Strip boxes, log phase; solid boxes, stationary phase. Student’s t-test used to determine statistical significance (p-value ≤0.005, **; ≤0.05, *). For log phase: ATCC974 vs. urea (p-value 0.0045) and NH_4_Cl (p-value 0.0008); urea vs. NH_4_Cl (p-value 0.0063). For stationary phase: ATCC974 vs. urea (p-value 0.0003) and NH_4_Cl (p-value 0.0006); urea vs. NH_4_Cl (p-value 0.0001). For urea: log vs. stationary (p-value 0.00007). For NH_4_Cl: log vs. stationary (p-value 0.0004). **(C)** Promoter activity of the carotenoid 3,4-desaturase *crtD* gene, quantified using a β-galactosidase reporter assay. The β-galactosidase (*bgaH*) of *H. alicantei* was fused to the promoter region of *crtD* to assess promoter activity. *H. volcanii* cells were grown in 0.18% (v/v) glycerol minimal media (GMM) supplemented with 10 mM urea or 10 mM NH_4_Cl as the nitrogen source or ATCC974 rich medium. From left to right, *H. volcanii* H26 carrying pJAM4483 with the *crtD* promoter region fused to *bgaH* (P*crtD-bgaH)*, β-galactosidase reporter plasmid pJAM2678 with the strong constitutive *H. cutirubrum* P2*rrnA* promoter (P2*rrnA-bgaH*), pJAM2714 with the removal of the promoter and the Shine Dalgarno (SD) sequence, and pJAM2715 with removal of only the P2_*rrnA*_ promoter. LP, log phase; SP, stationary phase. β-galactosidase activity (mU·mg^-1^ protein) was determined from triplicate cultures for each condition with 10 ug total protein. Data shown as mean ± SD (n = 3 biological replicates and 3 technical replicates). Statistical significance assessed using a Student’s t-test (p-value ≤0.005, **; ≤0.05, *). For *crtD* activity, during log phase growth activity differed significantly between urea and NH_4_Cl conditions (p 0.00035). Similarly, in stationary phase, a significant difference was observed between urea and NH_4_Cl (p-value 0.00005). Within nitrogen sources, significant differences were also detected between growth phases. For urea-grown cultures, log versus stationary phase comparisons yielded p-value 0.00035. For NH_4_Cl-grown cultures, log versus stationary phase comparisons resulted in p-value 0.00606. For ATCC-grown cultures, log versus stationary phase comparisons resulted in p-value 0.00019. For P2*rrnA* activity, during log phase growth activity did not differ significantly between urea and NH_4_Cl conditions (p ns). In stationary phase, a significant difference was observed between urea and NH_4_Cl (p-value 0.004). Within nitrogen sources, significant differences were also detected between growth phases. For urea-grown cultures, log versus stationary phase comparisons yielded p-value 0.0008. For NH_4_Cl-grown cultures, log versus stationary phase comparisons resulted in p-value 0.0016. For ATCC-grown cultures, log versus stationary phase comparisons resulted in p-value 0.0043. For no promoter or SD activity, all conditions were not significantly different. For SD only activity, the only condition that resulted in statistically significant for urea-grown cultures, log versus stationary phase comparisons yielded p-value 0.034. **(D)** Plate assay demonstrating *crtD* promoter region can drive high level expression of β-galactosidase. From left to right: P*crtD-bgaH,* P2*rrnA-bgaH,* empty *bgaH* plasmid devoid of promoter or SD sequence, pJAM2714 with the removal of the promoter and the SD sequence, and pJAM2715 with removal of only the P2_*rrnA*_ promoter. Hv-MM plates were supplemented with glycerol and 10 mM urea (left) vs.10 mM NH_4_Cl (right).

To investigate the molecular mechanism responsible for the increase in carotenoid content on urea vs. NH_4_Cl as a nitrogen source, the transcript abundance of the carotenoid biosynthetic *crtD* gene was evaluated using quantitative reverse transcription PCR (qRT-PCR). Primers were designed to specifically target the transcript of *crtD* (Figure 4A, green), a carotenoid 3,4-desaturase gene homolog associated with an early step in C_50_ carotenoid biosynthesis. Our hypothesis was that the abundance of *crtD*-specific transcripts is higher when cells are grown on urea compared to NH_4_Cl as the nitrogen source. To test this, *H. volcanii* H26 cells were grown in glycerol Hv-MM with either NH_4_Cl or urea as the nitrogen source. The cells were also grown on ATCC974 rich medium which includes peptides as the nitrogen source. qRT-PCR analysis of total RNA isolated from these cells revealed that *crtD*-specific transcript levels were highest in glycerol-grown cells when urea was used as the nitrogen source, compared to NH_4_Cl and growth in ATCC974 rich medium (Figure 4B). In glycerol grown cells, *crtD* transcript abundance was greater in stationary phase than in log phase regardless of the nitrogen source, with 9 to 10-fold increases in stationary phase observed on urea and NH_4_Cl, respectively. In contrast, *crtD* transcript levels remained low during both log and stationary phase in cells were grown on ATCC974 rich medium. Collectively, these findings suggest that *crtD* expression is strongly influenced by nitrogen source and growth phase.

### Transcription from the *crtD* promoter region is stimulated under urea

To further investigate the qRT-PCR results, a 237-bp DNA fragment encompassing the predicted *crtD* promoter was fused to a β-galactosidase reporter (*bgaH*) (Figure 4A, purple). To facilitate monitoring of gene expression and evaluation of regulatory responses associated with carotenoid production, a β-galactosidase reporter strain of *H. volcanii* was constructed. This construct was used to assess how nitrogen source and growth phase may affect transcription from the *crtD* promoter region and whether this promoter activity corresponded to the *crtD* transcript levels measured by qRT-PCR. Using β-galactosidase activity to monitor transcriptional activity (Figure 4C), the *crtD* promoter region was found to be highly active in driving *bgaH* expression. The transcriptional trends were consistent with the qRT-PCR results, with transcriptional activity of the *crtD* promoter region highest in stationary phase compared to log phase and in cells grown on glycerol with urea as the nitrogen source when compared to NH_4_Cl or ATCC974 medium. One notable difference was observed in ATCC974-grown cells, where the *crtD* promoter exhibited increased transcriptional activity during stationary phase relative to log phase, a pattern not reflected in the *crtD* transcript abundance monitored by qRT-PCR. Results with the controls were as anticipated including finding: i) the *bgaH* reporter fused to the *H. cutirubrum* P2*rrnA* promoter and pET-derived Shine-Dalgarno (ribosome binding) site displayed high level expression and ii-iii) the *bgaH* reporters lacking either the P2*rrnA* promoter alone or both the P2*rrnA* promoter and Shine-Dalgarno sequence were strongly reduced in expression (Figure 4C). These results reveal that the enhanced carotenoid production observed in the urea and crude glycerin feedstocks can be correlated with an increased transcription of carotenoid biosynthetic genes such as *crtD*.

### The *crtD* promoter region useful for biotechnology applications

The observation that *bgaH* transcription driven by the *crtD* promoter was comparable to that of the strong ribosomal P2*rrnA* promoter suggests that the *crtD* promoter is well suited for robust gene expression, including driving enzyme production in cultures containing waste feedstocks such as urea and glycerol. These findings also indicate that the *crtD* promoter may be useful in molecular biology applications as a reporter-driving element, enabling *bgaH* expression and β-galactosidase production that yields blue colonies in the presence of x-gal on agar plates. Consistent with this interpretation, cells expressing *bgaH* from the *crtD* promoter formed dark blue colonies with intensity and coloration comparable to those driven by the P2*rrnA* promoter (Figure 4D). In contrast, cells lacking these promoter regions produced pink to faintly blue colonies. One limitation of this blue/white screening plate assay is that the *crtD* promoter-driven expression reached levels that obscured differences between growth on urea verses NH_4_Cl. Taken together, these results demonstrate that the *crtD* promoter is a strong and versatile regulatory element, suitable for applications in *H. volcanii* requiring high-level gene expression on solid media or liquid culture, with nitrogen-source dependent differences in expression being more readily controlled and/or discerned in liquid culture than on agar plates.

## Discussion

Given that halophilic archaea preferentially metabolize glycerol (Nishihara et al., 1999; Sherwood et al., 2009; Williams et al., 2017), our results demonstrate that *H. volcanii* can use crude glycerin as an effective carbon source. This observation aligns with findings in mesophilic microorganisms such as *Methylorubrum extorquens* (Braunegg et al., 1999), *Komagataeibacter saccharivorans* (Gayathri and Srinikethan, 2018), *Rhodotorula glutinis* (Cutzu et al., 2013), and *Azotobacter vinelandii* (Yoshida et al., 2022). Notably, the low methanol content present in crude glycerin did not adversely affect *H. volcanii* growth compared to laboratory-grade glycerol (Figure 2A). Supporting these findings, glycerol kinase (GlpK) purified from *H. volcanii* retains catalytic efficiency and specific activity with crude glycerin as a substrate that is comparable to pure glycerol (Sanchez and Maupin-Furlow, 2025). Previous studies further demonstrate that haloarchaea such as *H. mediterranei* can convert crude glycerin into the biopolymer polyhydroxyalkanoate (PHA) (Atarés et al., 2024; Hermann-Krauss et al., 2013), underscoring the biotechnological potential of these microorganisms. Overall, these observations reinforce crude glycerin as a viable feedstock for carotenoid biosynthesis in halophilic archaea and other pigment-producing microorganisms.

Though this study focused on the utilization of crude glycerin as a sole carbon source, carotenoid optimization results indicate that substitution of urea for NH_4_Cl resulted in a deeper pink pigmentation at stationary phase (Figure 2B), thus resulting in higher carotenoid yield (Figure 3A). During cellular growth, cultures grown with NH_4_Cl occasionally exhibited biofilm formation, whereas biofilm development was not observed in urea-grown cultures (data not shown). Growth on reused crude glycerin–based medium (Figure 3B) was strongly dependent on trace element supplementation. Minimal supplementation resulted in low biomass (OD_600_ ≤ 0.37), whereas addition of glycerol, uracil, urea, KPO_4_, and trace elements significantly enhanced growth (OD_600_ 0.73 ± 0.06). Maximal growth was achieved only with combined trace element and vitamin (thiamine and biotin) supplementation (OD_600_ 0.95 ± 0.01). Thus media lacking trace elements failed to support robust growth, highlighting their necessity for sustained biomass accumulation.

To assess activity beyond exponential growth, glycerol was added at stationary phase (Figure 3C), resulting in increased carotenoid production and biomass compared to pre-supplementation levels. In hypersaline environments, compatible solutes stabilize proteins and cellular structures by maintaining osmotic balance (Ben-Amotz and Avron, 1973; Raymond et al., 2020; Reed et al., 1987). We hypothesize that glycerol could sustain redox metabolism by promoting NADH/NADPH generation through residual carbon flux or storage pathways, thereby supporting electron transport chain activity and maintenance energy production during stationary phase.

Although carotenoid composition was not directly resolved by HPLC in this study, prior reports indicate that halophilic carotenoid pools are dominated by bacterioruberin and its derivatives, with other carotenoids (*e.g.,* lycopene, β-carotene, phytoene) present at comparatively low abundance (Asker and Ohta, 1999; Hwang et al., 2024). The observed absorption maxima (371, 387, 472, 496, and 529 nm) are consistent with the characteristic spectral profile of bacterioruberin (Alvares and Furtado, 2021; Kesbiç et al., 2023), whereas alternative carotenoids display distinct absorbance features. Lycopene exhibits absorbance maxima at 446, 472, and 505 nm (Butnariu and Giuchici, 2011), while phytoene and phytofluene primarily absorb at 287 nm and 348–366 nm, respectively (Chemat-Djenni et al., 2010). Collectively, these observations support that the measured spectral profile is predominantly attributable to bacterioruberin and its derivatives.

Given the enhanced pigmentation, we analyzed transcriptional regulation of the carotenoid biosynthesis gene *crtD.* By qRT-PCR and β-galactosidase activity, we found supplementation with urea increased *crtD* transcript levels and activity, suggesting the involvement of a nitrogen-responsive transcriptional regulator (Figures 4B,C). These findings indicate that the *crtD* promoter is a tunable regulatory element for metabolic engineering. A β-galactosidase reporter system was used to quantify promoter activity due to its robustness and reliability in *H. volcanii* under high-salt conditions (Patenge et al., 2000; Gregor and Pfeifer, 2001). Future studies could leverage established expression systems already available in *H. volcanii,* including short-lived green fluorescent protein reporters (Davis et al., 2020) and colorimetric reporter platforms (Frecha et al., 2025), to enable real-time monitoring of promoter activity.

Urea supplementation has been associated with increased carotenoids production across diverse systems, including increased capsanthin (C_40_ carotenoid) and upregulation of carotenoid biosynthesis genes in a red pepper (Shen et al., 2024), as well as elevated β-carotene and torularhodin in *Rhodotorula* (Donzella et al., 2026). In additional, urea has been shown to promote cellular growth in *Chlamydomonas reinhardtii* (Rosa et al., 2023), and to enhance both biomass and carotenoid production in *Desmodesmus subspicatus* (Eze et al., 2022). However, the precise regulatory mechanism linking urea metabolism to carotenoid biosynthesis remains unresolved, and further studies will be required to directly establish the connection between halophilic *crtD* regulation and nitrogen metabolism. Additionally, by leveraging its strong induction under urea and glycerol-based growth medium, this promoter can be used to drive expression of heterologous or native biosynthetic genes that are not normally induced under these culture conditions. Thus, providing a flexible strategy to decouple biomass accumulation from product biosynthesis and expand the biotechnological utility of *H. volcanii*.

The *H. volcanii* genome encodes a urease complex comprising three structural subunits (HVO_0147 to HVO_0149) and four accessory proteins (HVO_0150 to HVO_0153) required for nickel incorporation into the active site, as well as a biotin-dependent carboxylase (Hartman et al., 2010). Urease hydrolyses urea into two ammonia (NH_3_) and one carbon dioxide (CO_2_) (Estiu and Merz, 2006), whereas NH_4_Cl supplies only one ammonium per molecule. The use of urea may thus offer osmotic and energetic advantages: urea introduces neutral compounds and releases NH_3_ intracellularly without contributing excess ionic load, whereas NH_4_Cl adds chloride ions that can disrupt ionic balance in high-salt environments. NH_3_ is subsequently protonated to NH_4_^+^, entering nitrogen assimilation via glutamine synthetase and glutamate dehydrogenase (Jiang et al., 2023; Li et al., 2012). Urea diffuses passively across membranes (Culham et al., 2001; Raunser et al., 2009; Wang et al., 2023), while NH_4_^+^ transport requires active ion pumps such as AmtB (Kim et al., 2012; Zheng et al., 2004), making urea energetically favorable for uptake. *H. volcanii* exhibits detectable urease activity in minimal medium (Schulze et al., 2020), consistent with these findings. Furthermore, urea offers practical benefits including higher nitrogen content (46% vs. 25% for NH_4_Cl), lower acidification potential, reduced production cost, and industrial scalability (Eze et al., 2022; Megda et al., 2019; Mohd Ibrahim et al., 2014; Zong et al., 2025).

In this study, urea supplementation alone increased pigmentation in *H. volcanii*, without genetic modification (Figure 3). Though, media and genetic enhancement of carotenoid yield have previously been achieved in other halophilic organisms (Table 4). Within halophilic archaea, *H. mediterranei* and *H. volcanii* via overexpression of lycopene pathway genes or mutation of the *lonB* protease, leading to hyperpigmented phenotypes (Cerletti et al., 2022; 2014; Zuo et al., 2018). Optimization strategies across diverse microorganisms, including *Sphingobium, E. coli, Rhodotorula, Yarrowia lipolytica*, and *Dunaliella salina* have successfully increased carotenoid yields (*e.g.,* β-carotene, lycopene and astaxanthin) using metabolic engineering and substrate adaptation (Choi et al., 2021; Cutzu et al., 2013; Iba et al., 2023; Jing et al., 2023; Lee et al., 2022; Liu et al., 2021; Wu et al., 2020). In parallel, halophilic archaea represent a promising microbial platform due to their inherent robustness under extreme stress conditions (Matarredona et al., 2024), enhanced antioxidant capacity (Ben Hamad Bouhamed et al., 2024), and the absences of a rigid cell wall, which facilitates ease of cell lysis (von Kügelgen et al., 2021). Additionally, their heterotrophic growth eliminates the requirement for photobioreactors for growth (Chen et al., 2023), all offering advantages compared to bacteria, yeast, and microalgae carotenoid production.

**TABLE 4:** This study in comparison with previous studies on halophilic archaea, with a focus on strategies aimed at enhancing bacterioruberin biosynthesis. Carotenoid production by halophilic archaeon was reported on a per-liter basis.

Organism	Strain	Optimization	Media	Bacterioruberin production maximum	Ref.
Minimal media					
*Haloferax volcanii*	H26	Compared nitrogen source (10 mM urea vs. NH_4_Cl); Supplemented crude glycerin cultures, with re-use of medium and additional crude glycerin (at 0.18% per supplement); Examined 0.1 L cultures	Hv-Min	0.90 ± 0.05 mg·L^-1^	This study
*Haloferax volcanii*	*Hv*LON3*	Compared *Hv*LON3 (LonB protease) mutant that displays hyperpigmentation vs. parent (H26); analyzed C_50_ carotenoid production	Hv-Min	-	Cerletti et al. (2014), Churio et al. (2022)
Complex media					
*Haloferax mediterranei*	R-4	Tested growth phase, glucose vs. yeast extract, starvation, inorganic salts, and carbon/nitrogen ratios; analyzed C_50_ carotenoid production; Examined 0.01 L cultures	Complex	3.76 mg·L^-1^	Giani et al. (2021)
*Haloferax mediterranei*	R-4	Used additional carbon sources, supplemented with industrial waste from the candy industry containing 0.5%–2.5% starch; analyzed C_50_ carotenoid production; Examined 0.01 L cultures	Complex	9.73 ± 0.186 mg·L^-1^	Giani et al. (2024)
*Haloarcula* sp. A15	-	Tested salt concentration, incubation temperature, pH, and carbon/nitrogen sources; Examined 0.1 L cultures	Complex	0.73 mg·L^-1^	Shahbazi et al. (2023)
*Halobacterium salinarum*	-	Isolated on DSC-97 growth medium from brine samples collected from solar saltern of Sfax City, Tunisia; analyzed C_50_ carotenoid production. Examined 0.1 L cultures	Complex	2.15 ± 0.39 mg·L^-1^	Ben Hamad Bouhamed et al. (2024)

* Bioengineered strain for carotenoid biosynthesis.

While the role of urea in archaeal metabolism remains underexplored, studies in algae and bacteria indicate that urea supplementation enhances both carotenoid accumulation and biomass yield. Urea catabolism releases bicarbonate, which feeds into carbon-concentrating mechanisms (CCM) and increases intracellular CO_2_, thereby stimulating growth and secondary metabolite production (Batista et al., 2019; Coleman et al., 2024; Rosa et al., 2023; Shi et al., 2000). Similar effects have been observed across taxa, including *Desmodesmus subspicatus, Flavobacterium multivorum, Phaffia rhodozyma, Rhodosporidium paludigenum, and Rhodotorula glutinis* (Bhosale and Bernstein, 2004; Cutzu et al., 2013; Eze et al., 2022; Kesava et al., 1998; Yimyoo et al., 2011)*.* Additionally, the dinoflagellate *Prorocentrum minimum* preferentially assimilates urea, with uptake rates exceeding those of nitrate and suppressing nitrate assimilation when both are available (Matantseva et al., 2016). Furthermore, nitrogen availability also influences polyhydroxyalkanoates (PHA) biosynthesis, as nutrient limitation redirects carbon flux from growth toward storage polymers. Under nitrogen starvation, excess carbon is converted into intracellular PHA granules (Surendran et al., 2020; Zhou et al., 2022).

To our knowledge, this study represents the first report of *H. volcanii* cultivated using crude glycerin as a primary carbon source. Establishing robust growth under these conditions provides a baseline medium for future metabolic optimization and genetic engineering strategies aimed at enhancing value-added bioproduct synthesis from industrial waste streams. Notably, nitrogen source optimization through substitution of NH_4_Cl with urea increased carotenoid production, demonstrating that targeted media refinement can substantially enhance pigment yield. Given that *H. volcanii* is a genetically robust and highly tractable archaeal model organism, this optimized crude glycerin-urea medium provides a strong platform for subsequent strain engineering and pathway enhancement efforts.

Collectively, these results identify urea as an effective nitrogen source that promotes biomass accumulation and high-level carotenoid production in *H. volcanii* grown on crude glycerin. When grown in urea and crude glycerin, an increased OD_600_ and carotenoid content are observed, even after normalization to the lowest OD_600_ value. Furthermore, this study establishes a cost-effective and environmentally sustainable strategy for valorizing crude glycerin, an industrial byproduct, through its conversion into high-value carotenoids. These improvements were achieved without genetic modification of the model organism, but rather through optimization of cultivation conditions and refinement of the carotenoid extraction protocol, resulting in increased bacterioruberin yield.

## Data Availability

The original contributions presented in the study are included in the article/Supplementary Material, further inquiries can be directed to the corresponding author.
