# Supplementary material for: Microbial conversion of biodiesel waste for carotenoid production
Source: Front Bioeng Biotechnol. 2026 Jun 29;14:1851919. doi: 10.3389/fbioe.2026.1851919 (PMC13358004; doi:10.3389/fbioe.2026.1851919)
Supplement: Supplementary file 1 [file Table1.DOCX]

**Supplemental Table 1**. Acetone extracted from carotenoid production of *H. volcanii* strain H26 grown on Hv-minimal media (Hv-MM) with carbon and nitrogen sources as indicated.

|  |  | mL of acetone extracted | | | |
| --- | --- | --- | --- | --- | --- |
| **3A** | **Culture condition** | **NH_4_Cl and crude glycerin** | **NH_4_Cl and glycerol** | **Urea and crude glycerin** | **Urea and glycerol** |
|  | mL yield | 5.6 ± 0.2 | 5.4 ± 0.2 | 5.6 ± 0.1 | 5.6 ± 0.1 |
|  |  |  |  |  |  |
| **3B** | **Culture condition** | **Starter culture** | **Reuse 1** | **Reuse 2** | **Reuse 3** |
|  | mL yield | 5.2 ± 0.3 | 5.6 ± 0.1 | 5.2 ± 0.3 | 5.7 ± 0.3 |
|  |  |  |  |  |  |
| **3C** | **Culture condition** | **No additional glycerol** | **Additional glycerol** | **No additional crude glycerin** | **Additional crude glycerin** |
|  | mL yield | 5.2 ± 0.3 | 7.5 ± 2.2 | 4.6 ± 0.4 | 7.7 ± 3.0 |

Datasets supporting Figure 3 panels A, B, and C as indicated on the left; details regarding carbon and nitrogen supplementation are described in Figure 3. The final extraction volume for the optimized carotenoid combined strategies of using the spent medium (reuse 1) and supplementing stationary-phase cultures with additional crude glycerin was 10.47 ± 1.55 mL acetone.
