# Supplementary figures and images for: Microbial conversion of biodiesel waste for carotenoid production
Source: Front Bioeng Biotechnol. 2026 Jun 29;14:1851919. doi: 10.3389/fbioe.2026.1851919 (PMC13358004; doi:10.3389/fbioe.2026.1851919)

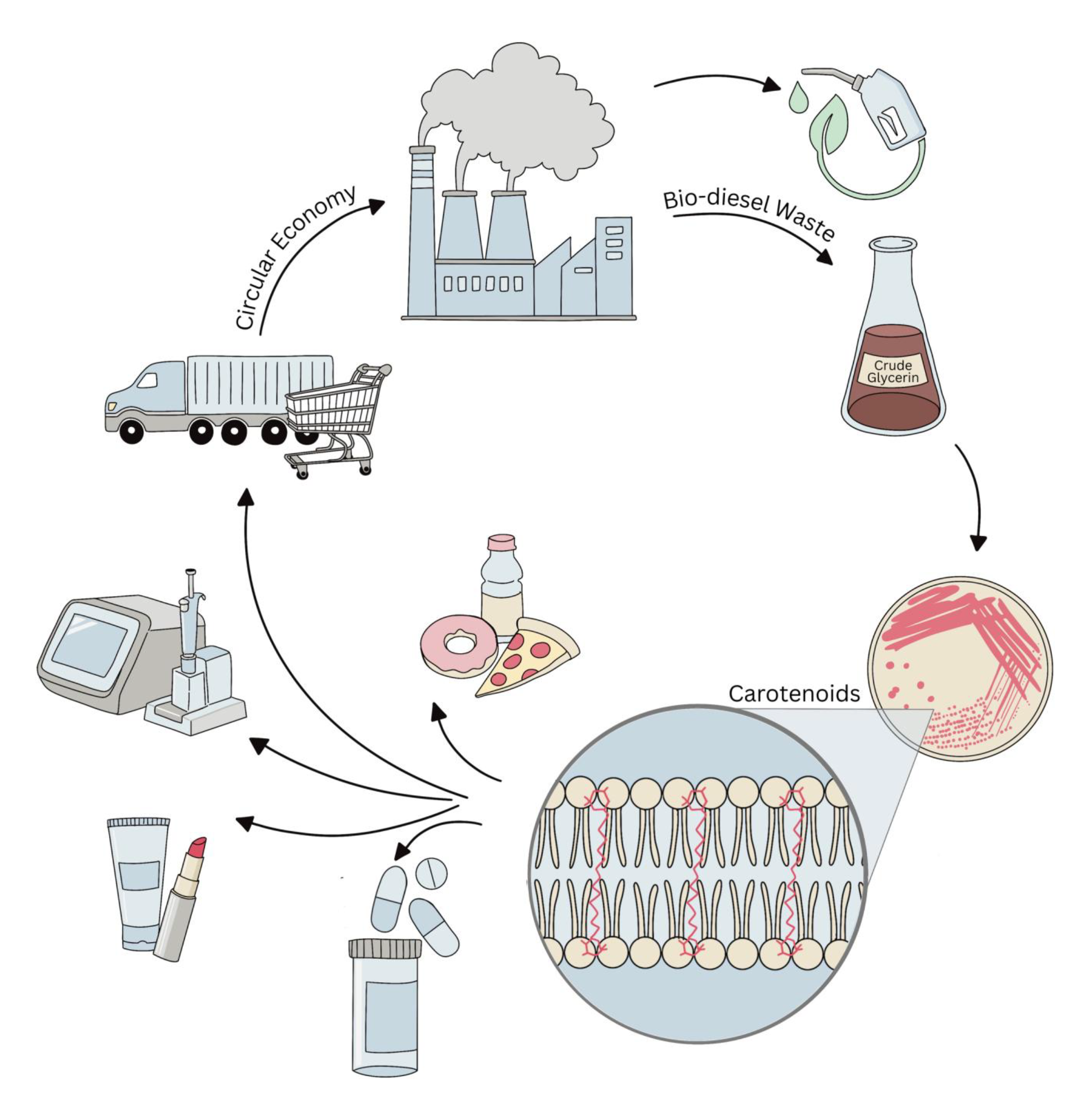

Supplement: Supplementary file 2 [file Image1.TIF]
