# Supplementary material for: Microbial conversion of biodiesel waste for carotenoid production
Source: Front Bioeng Biotechnol. 2026 Jun 29;14:1851919. doi: 10.3389/fbioe.2026.1851919 (PMC13358004; doi:10.3389/fbioe.2026.1851919)
Supplement: Supplementary file 3 [file Table2.docx]

**Supplemental Table 2**. Carotenoid production of *H. volcanii* strain H26 grown on Hv-minimal media (Hv-MM) with carbon and nitrogen sources as indicated.

|  |  | Carotenoid (mg·L^-1^) recovered from 100 mL culture | | | |
| --- | --- | --- | --- | --- | --- |
| **3A** | **Culture condition** | **NH_4_Cl and crude glycerin** | **NH_4_Cl and glycerol** | **Urea and glycerol** | **Urea and crude glycerin** |
|  | Overall yield | 11.8 ± 3.9 | 11.4 ± 1.9 | 30.4 ± 1.3 | 32.2 ± 2.5 |
|  | Normalized | 10.0 ± 2.4 | 9.5 ± 1.7 | 20.8 ± 1.4 | 22.1 ± 2.0 |
|  |  |  |  |  |  |
| **3B** | **Culture condition** | **Starter culture** | **Reuse 1** | **Reuse 2** | **Reuse 3** |
|  | Overall yield | 32.8 ± 0.4 | 50.0 ± 3.2 | 15.5 ± 2.8 | 14.3 ± 3.8 |
|  | Normalized | 21.0 ± 0.3 | 31.1 ± 3.1 | 10.8 ± 2.0 | 14.2 ± 3.8 |
|  |  |  |  |  |  |
| **3C** | **Culture condition** | **No additional glycerol** | **Additional glycerol** | **No additional crude glycerin** | **Additional crude glycerin** |
|  | Overall yield | 40.3 ± 2.8 | 76.0 ± 8.2 | 37.2 ± 2.0 | 68.5 ± 3.8 |
|  | Normalized | 34.7 ± 7.2 | 63.1 ± 8.0 | 28.0 ± 2.8 | 68.4 ± 4.1 |

Datasets supporting Figure 3 panels A, B, and C as indicated on the left; details regarding carbon and nitrogen supplementation are described in Figure 3.
